# Age-related associations between the CALLY index and advanced cardiovascular-kidney-metabolic (CKM) syndrome: Insights from NHANES population data

**DOI:** 10.1097/MD.0000000000047718

**Published:** 2026-03-13

**Authors:** Yi Liu, Yong Li

**Affiliations:** a Department of Urology, The Second Affiliated Hospital, Hengyang Medical School, University of South China, Hengyang, China.

**Keywords:** CALLY index, CKM syndrome, inflammation, NHANES, nutritional

## Abstract

Cardiovascular disease, chronic kidney disease, and metabolic syndrome often co-occur, forming a complex disorder known as cardiovascular-kidney-metabolic (CKM) syndrome. Identifying biomarkers that reflect inflammation, nutrition, and immune function is crucial for effective risk assessment. The C-reactive protein-albumin-lymphocyte (CALLY) index, which integrates these factors, has not been explored in the context of CKM syndrome. Data from 9 National Health and Nutrition Examination Survey cycles (2001–2018) were used, including 11,866 adults aged 20 years and older. The CALLY index was calculated as (albumin × lymphocyte count) ÷ C-reactive protein and then log-transformed. CKM syndrome was categorized into stages 0 to 4. Multivariable logistic regression and smoothing splines were used to assess associations between the CALLY index and advanced CKM (stages 3 and 4), with subgroup analyses by age and other variables. A higher log-transformed CALLY index was associated with a lower likelihood of advanced CKM (odds ratio per unit increase: 0.90; 95% confidence interval: 0.86–0.95). Individuals in the top tertile had a 21% lower odds of advanced CKM than those in the bottom tertile (odds ratio: 0.79; 95% confidence interval: 0.67–0.93). A threshold effect was observed in individuals under 60 years, with protective effects increasing below a CALLY value of 455 units. The CALLY index is inversely associated with advanced CKM syndrome, reflecting systemic inflammation, nutrition, and immune status. Its age-dependent associations suggest opportunities for early detection and targeted interventions, especially in younger adults. Validation in prospective studies is needed.

## 1. Introduction

Cardiovascular disease (CVD), chronic kidney disease (CKD), and metabolic syndrome (MS) frequently co-occur, forming an interrelated constellation of pathologies that collectively elevate cardiometabolic risk.^[1,2]^ This clustering is especially common among individuals with type 2 diabetes mellitus (T2DM), highlighting the overlapping pathophysiological pathways that worsen patient outcomes.^[3]^ Notably, the simultaneous presence of CKD and T2DM markedly amplifies cardiovascular morbidity and mortality, reflecting the synergistic exacerbation inherent in these disorders. In response to this clinical complexity, the American Heart Association introduced the concept of cardiovascular-kidney-metabolic (CKM) syndrome, which frames these interdependent dysfunctions as a singular systemic disorder characterized by progressive impairment across cardiovascular, renal, and metabolic domains.^[4,5]^ The CKM syndrome is stratified into stages 0 through 4 based on composite metrics assessing multiorgan involvement and severity, facilitating a more nuanced understanding of disease progression and risk.^[6]^

The development of CKM syndrome arises from multiple intertwined mechanisms, including persistent systemic inflammation, increased oxidative stress, impaired endothelial function, and activation of neurohormonal pathways.^[7,8]^ Collectively, these processes drive the advancement of each separate condition – CVD, CKD, and metabolic abnormalities – while also amplifying their combined harmful effects through mutual reinforcement.^[9,10]^ Identifying reliable biomarkers that reflect this multifaceted pathophysiological state is critical for early diagnosis, risk stratification, and monitoring therapeutic responses within the CKM framework. Among potential biomarkers, the combined assessment of inflammatory, nutritional, and immunological parameters has gained increasing attention. The C-reactive protein-albumin-lymphocyte (CALLY) index, a recently developed composite biomarker, synthesizes 3 pivotal dimensions of systemic health: inflammation, nutritional status, and immune competence.^[11,12]^ Preliminary studies have demonstrated its prognostic utility across a variety of clinical contexts, including malignancies, chronic inflammatory diseases, and cardiovascular disorders.^[13–16]^ However, its application in the setting of CKM syndrome remains unexplored. Considering the complex interplay among cardiovascular, kidney, and metabolic impairments in CKM syndrome, exploring how the CALLY index correlates with disease severity or development could yield valuable insights. Utilizing data from the National Health and Nutrition Examination Survey (NHANES) database enables an extensive population-based evaluation of this association, leveraging comprehensive biochemical and clinical parameters. Clarifying whether the CALLY index can serve as an effective composite biomarker for identifying high-risk CKM patients may inform personalized risk assessment and guide therapeutic interventions aimed at mitigating the systemic burden of this syndrome.^[17]^

## 2. Methods

### 2.1. Study population

**Figure 1.**
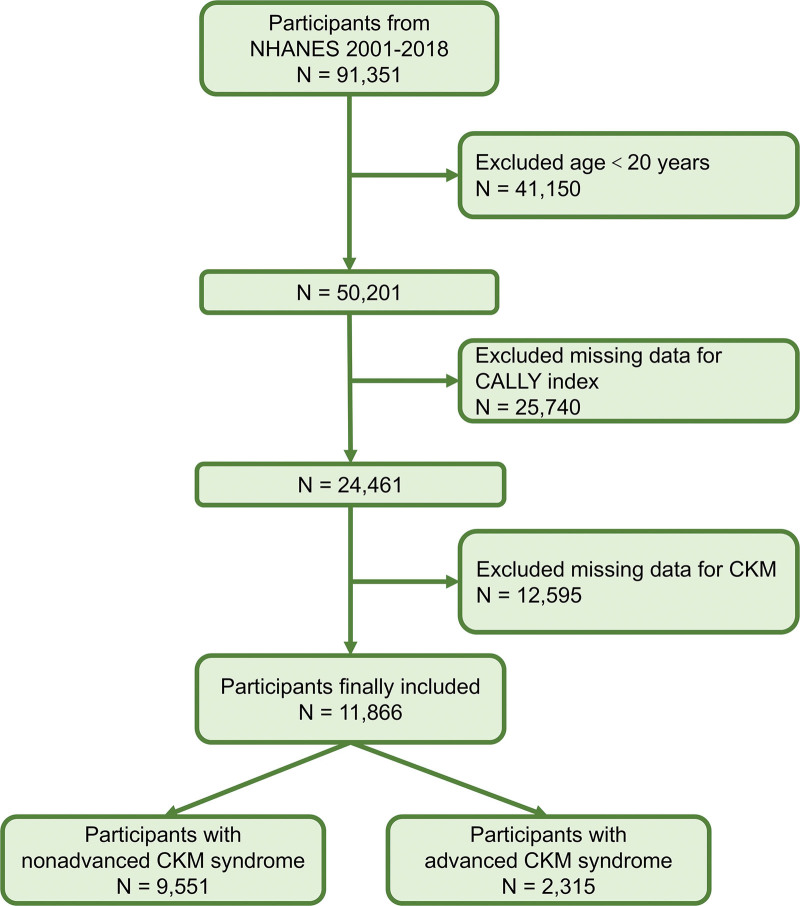
Flowchart illustrating the participant selection process. Classification criteria: nonadvanced CKM syndrome includes stages 0 to 2; advanced CKM syndrome includes stages 3 and 4. CALLY index = C-reactive protein-albumin-lymphocyte index, CKM = cardiovascular-kidney-metabolic, NHANES = National Health and Nutrition Examination Survey.

This study analyzed publicly available data from the NHANES, a cross-sectional dataset that uses a stratified, multistage probability sampling design to generate representative health and nutrition estimates for the civilian, noninstitutionalized US population.^[18,19]^ Ethical approval for NHANES was granted by the National Center for Health Statistics Research Ethics Review Board, and all participants provided informed consent before taking part, ensuring adherence to accepted ethical standards.^[20]^ Our analysis incorporated data spanning 9 survey cycles from 2001 through 2018. To ensure methodological rigor and target the adult population typically affected by CKM syndrome, individuals younger than 20 years (n = 41,150) were excluded due to differing disease incidence patterns and potentially distinct pathophysiological mechanisms in younger cohorts. Additionally, individuals missing key laboratory parameters essential for calculating the CALLY index – specifically serum albumin, lymphocyte count, or C-reactive protein (CRP) measurements – were omitted (n = 25,740). Participants lacking sufficient data to ascertain CKM syndrome status based on clinical and biochemical criteria were also excluded (n = 12,595). Following these sequential exclusions, the final analytic sample encompassed 11,866 adults (refer to Fig. 1 for the detailed participant flow diagram).

### 2.2. Calculation of the CALLY index

The CALLY index was operationalized utilizing the validated algorithm introduced by Iida et al,^[11]^ integrating systemic inflammation, nutritional status, and immunological competence into a single composite biomarker. The index is computed as follows:


$$\begin{array}{l} CALLY\;index=\frac{albumin\;\left(g/L\right)\times lymphocyte\;\left(1000\;cells/\mu L\right)}{CRP\;\left(mg/dL\right)} \end{array}$$

Serum albumin, lymphocyte count, and CRP concentrations were measured using automated hematology analyzers and immunoassays consistent across NHANES cycles. To improve data normality and mitigate the influence of outliers, the CALLY index underwent natural logarithmic transformation prior to analysis (log-transformed CALLY). This transformation optimized compliance with linear regression assumptions, enhanced model robustness, and facilitated interpretation by stabilizing variance and improving linearity in associations, including those with clinical outcomes such as CKM syndrome severity.

### 2.3. Definition and staging of CKM syndrome

Classification and staging of CKM syndrome were grounded in the framework promulgated by the American Heart Association Presidential Advisory, further refined according to the staging schema developed by Aggarwal et al.^[6]^ This approach was adapted to align with biochemical and clinical data available through NHANES cycles 2001 to 2018 to ensure accurate representation of disease staging in this population. Demographic variables such as age, sex, and race/ethnicity were self-reported during structured interviews, and anthropometric data, including weight, height, and waist circumference, were obtained via standardized NHANES measurement protocols. Metabolic abnormalities – comprising prediabetes, diabetes mellitus, hypertension, dyslipidemia (notably hypertriglyceridemia), and MS – were delineated following established cutoffs of validated biomarkers such as glycated hemoglobin (HbA1c), blood pressure readings, and lipid profiles. The CKM syndrome staging ranges from 0 (no or minimal impairment) to 4 (severe multiorgan dysfunction), reflecting escalating cardiovascular and renal risk profiles. Metabolic risk factors were incorporated alongside anthropometric indices including waist circumference, with ethnicity-specific cut points implemented, particularly for Asian subpopulations, to accommodate known variations in body composition and cardiometabolic risk profiles. Comprehensive staging criteria are provided in Table S1, Supplemental Digital Content, https://links.lww.com/MD/R490. For analytical clarity, participants were classified into 2 groups: advanced CKM (stages 3 and 4) and nonadvanced CKM (stages 0 through 2).^[21,22]^

### 2.4. Covariables

A wide array of potential confounding factors spanning demographic, socioeconomic, behavioral, and clinical domains were included in the multivariable models. These variables comprised age, sex, race/ethnicity, level of education, poverty-income ratio (PIR), smoking habits, alcohol consumption patterns, and comorbid conditions such as chronic obstructive pulmonary disease (COPD). Laboratory assessments covered HbA1c, liver enzymes (alanine aminotransferase [ALT] and aspartate aminotransferase [AST]), and lipid profile components, such as total cholesterol, low-density lipoprotein cholesterol (LDL-C), and high-density lipoprotein cholesterol (HDL-C). While behavioral variables were based on self-report and therefore subject to potential recall or reporting bias, biomarker and anthropometric measurements were obtained following rigorous NHANES protocols, supporting the reliability and standardization of data throughout survey periods.

### 2.5. Statistical analysis

Descriptive comparisons of demographic, clinical, and laboratory variables across tertiles of the natural log-transformed CALLY index were conducted using one-way analysis of variance for continuous variables with normal distributions, while chi-square tests were applied to categorical data. To explore associations between the CALLY index and the presence of advanced CKM syndrome, multivariable logistic regression models were constructed. Covariates were selected based on their established biological plausibility and prior literature, indicating their association with both CKM syndrome and the components of the CALLY index. We employed 3 hierarchical models to control for confounding factors: model 1: unadjusted; model 2: adjusted for demographic factors (age, sex, and race/ethnicity); and model 3: fully adjusted for age, sex, race/ethnicity, education level, PIR, smoking behavior, alcohol consumption, height, HbA1c, ALT, AST, COPD, triglycerides, total cholesterol, HDL-C, and LDL-C. The index was examined both as a continuous measure and categorized into tertiles, with trend analyses performed to evaluate potential linear dose-response effects. Subgroup analyses were carried out based on sex, age groups, racial/ethnic backgrounds, PIR, education level, smoking habits, alcohol intake, and COPD status to assess the consistency and strength of these relationships. Interaction terms were incorporated to determine whether effect modification existed among these factors. Additionally, smooth curve fittings were initially performed to address the potential nonlinearity of the association between the CALLY index and advanced CKM syndrome. To detect any threshold effects, a 2-piecewise logistic regression model was employed to calculate the inflection point using a recursive algorithm. A log-likelihood ratio test was conducted to compare the standard 1-line logistic regression model with the 2-piecewise logistic regression model, determining whether the relationship was linear or nonlinear. All statistical procedures were executed using R software (version 4.3.3; The R Foundation for Statistical Computing, Vienna, Austria) alongside EmpowerStats (version 6.0; X&Y Solutions, Inc., Boston). The results achieving a 2-sided *P*-value below .05 were considered statistically significant.

## 3. Results

### 3.1. Baseline characteristics

The analyzed sample included 11,866 individuals aged 20 years and older, with an average age of approximately 49.8 years (±18.5 years). Females represented 51.63% of the sample, while non-Hispanic Whites constituted 50.39%. The mean CALLY index was 888.26 ± 1563.24, with distribution across tertiles as follows: T1, 109.35 ± 53.76; T2, 390.95 ± 125.39; and T3, 1885.31 ± 1487.09. Approximately 20% of participants were classified as having advanced CKM syndrome. Individuals classified within the highest tertile of the CALLY index were more often male and had a greater representation of those identifying as Other races, compared with participants in the lowest tertile. This group also exhibited lower prevalences of hypertension, diabetes, and COPD. Notably, increasing CALLY index was associated with lower glycemic markers (HbA1c), reduced hepatic enzymes (ALT and AST), and decreased lipid parameters, including triglycerides, total cholesterol, and LDL-C. Conversely, higher CALLY values correlated positively with HDL-C levels and height (Table 1).

**Table 1:** Baseline characteristics of the study population according to tertiles of CALLY index.

Characteristics	CALLY index			*P*-value
	T1 (N = 3955)	T2 (N = 3948)	T3 (N = 3963)	
Age (yr)	51.99 ± 18.69	51.39 ± 17.97	46.05 ± 18.21	<.001
Sex (%)				<.001
Male	37.04	49.90	58.16	
Female	62.96	50.10	41.84	
Race/ethnicity (%)				<.001
Non-Hispanic White	50.09	50.84	50.24	
Mexican American	19.57	21.20	18.47	
Non-Hispanic Black	21.29	17.00	17.99	
Other races	9.05	10.97	13.30	
Education level (%)				<.001
Some college or above	51.22	51.71	50.89	
High school	23.30	22.58	23.42	
Less than high school	25.48	25.71	25.70	
PIR (%)				<.001
<1.3	28.01	28.02	28.25	
≥1.3, <3.5	43.40	42.22	43.70	
≥3.5	28.59	29.76	28.05	
Smoke (%)				<.001
Never smoker	50.42	51.87	53.97	
Former smoker	28.98	25.81	23.90	
Current smoker	20.61	22.32	22.13	
Alcohol use (%)				<.001
Never	15.50	12.66	11.46	
Former	23.01	19.63	15.52	
Mild	33.43	37.41	38.88	
Moderate	12.09	12.41	13.25	
Heavy	15.98	17.88	20.89	
BMI (kg/m^2^)				<.001
<25	16.69	24.72	46.98	
≥25, <30	31.96	40.30	37.47	
≥30	51.35	34.98	15.54	
CKD (%)				<.001
No	75.40	81.41	86.83	
Yes	24.60	18.59	13.17	
Hypertension (%)				<.001
No	50.90	57.17	68.16	
Yes	49.10	42.83	31.84	
Diabetes mellitus (%)				<.001
No	76.61	81.76	87.36	
Yes	23.39	18.24	12.64	
COPD (%)				<.001
No	94.06	95.67	96.52	
Yes	5.94	4.33	3.48	
CKM syndrome (%)				<.001
Nonadvanced stages	74.92	80.22	86.32	
Advanced stages	25.08	19.78	13.68	
ALT (IU/L)	25.86 ± 49.34	26.59 ± 18.90	25.00 ± 15.49	<.001
AST (IU/L)	26.01 ± 35.90	25.97 ± 17.10	25.52 ± 12.71	<.001
Triglyceride (mg/dL)	151.22 ± 98.33	150.00 ± 113.59	124.95 ± 110.76	<.001
Total cholesterol (mg/dL)	199.36 ± 42.82	202.24 ± 43.20	192.70 ± 39.23	<.001
HDL-cholesterol (mg/dL)	52.65 ± 16.24	52.91 ± 15.49	56.08 ± 16.44	<.001
LDL-cholesterol (mg/dL)	116.78 ± 35.42	120.09 ± 36.06	112.71 ± 33.21	<.001
HbA1c (%)	5.80 ± 1.16	5.69 ± 1.05	5.50 ± 0.79	<.001
Height (cm)	165.80 ± 9.72	167.78 ± 10.15	169.34 ± 10.16	<.001
CALLY index	109.35 ± 53.76	390.95 ± 125.39	1885.31 ± 1487.09	<.001

Data are presented as mean ± SD for continuous variables and frequency (percentage) for categorical variables. *P*-values were calculated using one-way analysis of variance (ANOVA) for continuous variables and chi-square tests for categorical variables.

ALT = alanine aminotransferase, AST = aspartate aminotransferase, BMI = body mass index, CALLY index = C-reactive protein-albumin-lymphocyte index, CKD = chronic kidney disease, CKM = cardiovascular-kidney-metabolic, COPD = chronic obstructive pulmonary disease, HbA1c = hemoglobin A1c, HDL-cholesterol = high-density lipoprotein cholesterol, LDL-cholesterol = low-density lipoprotein cholesterol, PIR = poverty-income ratio.

### 3.2. Relationship between log-transformed CALLY index and advanced CKM syndrome

**Figure 2.**
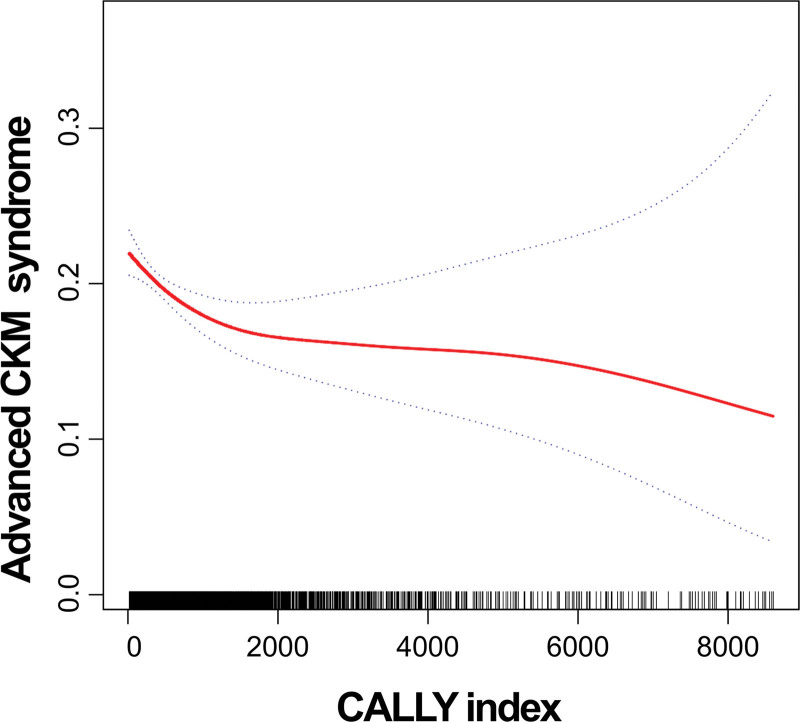
Nonlinear relationship between CALLY index and advanced CKM syndrome. A generalized additive model revealed a significant nonlinear relationship, identifying an inflection point at a CALLY value of 117.24. Below this threshold, higher CALLY index values were significantly associated with reduced risk of advanced CKM syndrome, whereas above the inflection point, the association plateaued and was no longer statistically significant. CALLY index = C-reactive protein-albumin-lymphocyte index, CKM = cardiovascular-kidney-metabolic.

After controlling for age, sex, race/ethnicity, education level, PIR, smoking status, alcohol consumption, height, HbA1c, ALT, AST, COPD, and lipid profiles (triglycerides, total cholesterol, HDL-C, and LDL-C), multivariable logistic regression models indicated an inverse relationship between the natural log-transformed CALLY index and the presence of advanced CKM syndrome. More specifically, each 1-unit increase in log-transformed CALLY corresponded to approximately a 10% decrease in the odds of advanced CKM (odds ratio [OR]: 0.90; 95% confidence interval [CI]: 0.86–0.95). When participants were grouped by tertiles, those in the highest tertile had a 21% reduced likelihood of advanced CKM relative to individuals in the lowest tertile (OR: 0.79; 95% CI: 0.67–0.93; Tables 2 and S2, Supplemental Digital Content, https://links.lww.com/MD/R490). Nonlinear patterns emerged from smoothing spline analyses, suggesting a complex association between the CALLY index and advanced CKM status (Fig. 2). These findings were supported by 2-piecewise logistic regression, which confirmed significant nonlinearity (*P* < .001, log-likelihood ratio test; Table 3). Specifically, for CALLY values below the cutoff point of 117.24, a notable inverse association with advanced CKM risk was observed (OR: 0.9540; 95% CI: 0.9285–0.9801), whereas above this threshold, the association was not statistically significant (OR: 0.9992; 95% CI: 0.9984–1.0000). At lower CALLY index values – indicative of heightened systemic inflammation, poorer nutritional status, and compromised immune function – the risk of advanced CKM increases sharply.

**Table 2:** Association between log-transformed CALLY index and advanced CKM syndrome.

Exposure	Model 1 (OR [95% CI]) *P*-value	Model 2 (OR [95% CI]) *P*-value	Model 3 (OR [95% CI]) *P*-value
Log-transformed CALLY	0.77 (0.74–0.79) <.0001	0.81 (0.78–0.85) <.0001	0.90 (0.86–0.95) .0001
Log-transformed CALLY classification			
Tertile 1	Reference	Reference	Reference
Tertile 2	0.74 (0.66–0.82) <.0001	0.69 (0.60–0.79) <.0001	0.83 (0.72–0.96) .0117
Tertile 3	0.47 (0.42–0.53) <.0001	0.58 (0.50–0.67) <.0001	0.79 (0.67–0.93) .0048

Model 1: no covariates were adjusted.

Model 2: age, sex, and race were adjusted.

Model 3: age, sex, race, education level, PIR, smoking behavior, drinking behavior, height, HbA1c, ALT, AST, COPD, triglyceride, total cholesterol, HDL-cholesterol, and LDL-cholesterol were adjusted.

ALT = alanine aminotransferase, AST = aspartate aminotransferase, CALLY index = C-reactive protein-albumin-lymphocyte index, CI = confidence interval, CKM = cardiovascular-kidney-metabolic, COPD = chronic obstructive pulmonary disease, HbA1c = hemoglobin A1c, HDL-cholesterol = high-density lipoprotein cholesterol, LDL-cholesterol = low-density lipoprotein cholesterol, OR = odds ratio, PIR = poverty-income ratio.

**Table 3:** Results of 2-piecewise logistic regression model.

Outcome: advanced CKM syndrome (dichotomy)
Risk factor of interest: CALLY (index, dimensionless)
Inflection point (K) of CALLY: 117.24
Effect size OR (95% CI) for CALLY (per 10 U) < K 117.24 0.9540 (0.9285–0.9801)
Effect size OR (95% CI) for CALLY (per 10 U) ≥ K 117.24 0.9992 (0.9984–1.0000)
*P* for log-likelihood ratio test < .001

Two-piecewise logistic regression model was used to calculate the threshold effect of the CALLY. If the log-likelihood ratio test <0.05, it means the 2-piecewise logistic regression model is superior to the single-line logistic regression model.

Adjustment model was adjusted for age, sex, race, education level, PIR, smoking behavior, drinking behavior, height, HbA1c, ALT, AST, COPD, triglyceride, total cholesterol, HDL-cholesterol, and LDL-cholesterol.

ALT = alanine aminotransferase, AST = aspartate aminotransferase, CALLY index = C-reactive protein-albumin-lymphocyte index, CI = confidence interval, CKM = cardiovascular-kidney-metabolic, COPD = chronic obstructive pulmonary disease, HbA1c = hemoglobin A1c, HDL-cholesterol = high-density lipoprotein cholesterol, LDL-cholesterol = low-density lipoprotein cholesterol, OR = odds ratio, PIR = poverty-income ratio.

### 3.3. Subgroup analyses

The inverse relationship observed between the natural log-transformed CALLY index and the likelihood of advanced CKM syndrome was examined within diverse demographic and behavioral subgroups to assess the robustness and potential effect modification of this association. Variables considered included age groups, sex, racial/ethnic categories, PIR, educational background, smoking status, alcohol consumption patterns, and presence of COPD (see Table 4). Notably, age emerged as the only factor significantly modifying the association (interaction *P* < .0001). Specifically, in adults younger than 60 years, a more pronounced protective influence was identified, with each incremental unit increase in log-transformed CALLY associated with an approximate 25% reduction in odds of advanced CKM syndrome. Conversely, among those aged 60 and older, the magnitude of risk attenuation per unit rise in log-transformed CALLY was less marked, estimated at 9%. By contrast, other subgroup analyses did not reveal statistically significant effect modification; the inverse association between higher CALLY index levels and advanced CKM remained consistent, regardless of sex, race/ethnicity, socioeconomic indicators, smoking or drinking behaviors, and COPD status (all *P* > .05).

**Table 4:** Subgroup analysis of the association between log-transformed CALLY index and advanced CKM syndrome.

Subgroup	OR (95% CI)	*P* for interaction
Sex		.1432
Female	0.85 (0.79–0.92)	
Male	0.92 (0.86–0.99)	
Age		<.0001
<60 yr	0.75 (0.70–0.82)	
≥60 yr	0.91 (0.86–0.96)	
Race/ethnicity		.7145
Non-Hispanic White	0.87 (0.82–0.93)	
Mexican American	0.94 (0.82–1.07)	
Non-Hispanic Black	0.91 (0.82–1.02)	
Other races	0.86 (0.73–1.01)	
PIR		.0977
<1.3	0.85 (0.77–0.93)	
≥1.3, <3.5	0.87 (0.81–0.94)	
≥3.5	0.97 (0.88–1.07)	
Smoke		.4628
Never smoker	0.88 (0.82–0.95)	
Former smoker	0.92 (0.85–1.00)	
Current smoker	0.85 (0.76–0.95)	
Alcohol use		.8850
Never	0.86 (0.75–0.98)	
Former	0.87 (0.79–0.95)	
Mild	0.90 (0.83–0.98)	
Moderate	0.95 (0.80–1.13)	
Heavy	0.89 (0.76–1.04)	
Education level		.6687
Some college or above	0.89 (0.82–0.96)	
High school	0.92 (0.83–1.02)	
Less than high school	0.87 (0.80–0.94)	
COPD		.8185
No	0.89 (0.84–0.94)	
Yes	0.91 (0.77–1.06)	

Age, sex, race, education level, PIR, smoking behavior, drinking behavior, height, HbA1c, ALT, AST, COPD, triglyceride, total cholesterol, HDL-cholesterol, and LDL-cholesterol were adjusted. In the subgroup analyses, the model is not adjusted for the stratification variable itself. The odds ratios (ORs) were derived from separate models for each subgroup, fully adjusted for all covariates (except the stratification variable itself). *P*-values for interaction were calculated using likelihood ratio tests.

ALT = alanine aminotransferase, AST = aspartate aminotransferase, CALLY index = C-reactive protein-albumin-lymphocyte index, CI = confidence interval, CKM = cardiovascular-kidney-metabolic, COPD = chronic obstructive pulmonary disease, HbA1c = hemoglobin A1c, HDL-cholesterol = high-density lipoprotein cholesterol, LDL-cholesterol = low-density lipoprotein cholesterol, OR = odds ratio, PIR = poverty-income ratio.

### 3.4. Nonlinear relationship of CALLY index (per 10 units) and advanced CKM syndrome in different age groups

**Figure 3.**
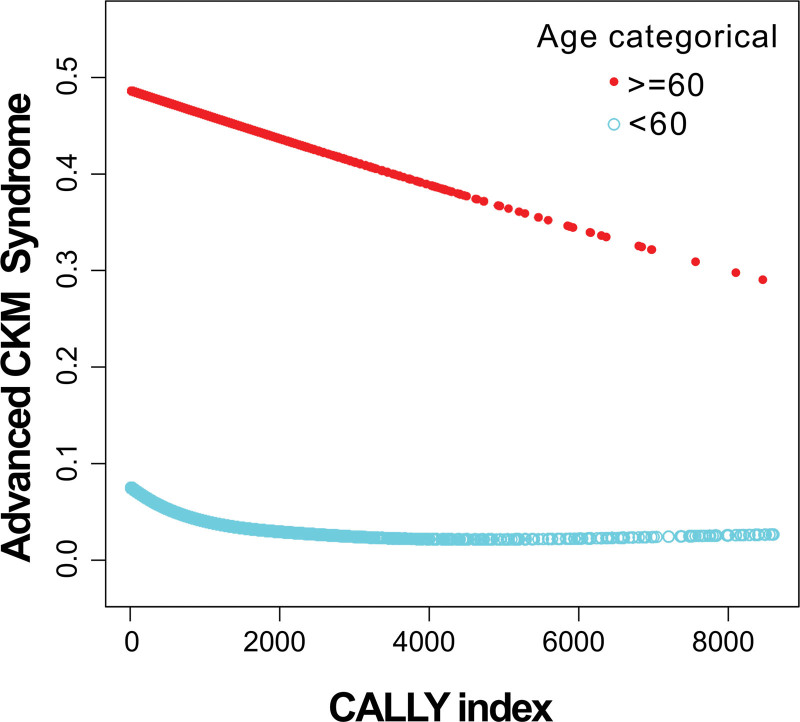
Nonlinear association between the CALLY index and advanced CKM syndrome among participants younger than 60 years. A generalized additive model identified a significant nonlinear relationship with an inflection point at a CALLY value of 455. Below this threshold, higher CALLY index values were strongly associated with a decreased risk of advanced CKM syndrome, while above the inflection point, the association plateaued and lost statistical significance. By contrast, among participants aged 60 years and older, the relationship between the CALLY index and advanced CKM syndrome was linear and modest in magnitude. CALLY index = C-reactive protein-albumin-lymphocyte index, CKM = cardiovascular-kidney-metabolic.

Generalized additive model analyses did not identify nonlinear associations when stratified by sex, race, PIR, smoking, or alcohol use. However, age-stratified analyses revealed distinct nonlinear relationships. For individuals younger than 60 years, there was a notable nonlinear association characterized by a discernible inflection point near a CALLY index value of 455 (Fig. 3). Below this cutoff, each 10-unit increment in the CALLY index corresponded to a modest yet statistically significant decrease in the risk of advanced CKM syndrome (OR: 0.9884; 95% CI: 0.9808–0.9960). Above this threshold, the relationship leveled off, with no significant further reduction in risk detected (OR: 0.9987; 95% CI: 0.9972–1.0002; Table 5). Conversely, in the subgroup aged 60 years and older, a linear logistic regression model provided a better fit than a 2-piecewise model (*P* for log-likelihood ratio test = .054), indicating a steady but less pronounced inverse association between the CALLY index and advanced CKM syndrome (OR: 0.9988; 95% CI: 0.9979–0.9997).

**Table 5:** The threshold effect of CALLY index (per 10 U) on advanced CKM syndrome stratified by age was analyzed using a 2-part linear regression model.

	Age	
	<60 yr	≥60 yr
Fitting by standard linear model		
OR (95% CI)	0.9976 (0.9963–0.9990)	0.9988 (0.9979–0.9997)
*P*-value	<.0004	.0088
Fitting by 2-piecewise linear model		
Inflection point (K)	455	109.33
OR1 (<K)	0.9884 (0.9808–0.9960)	0.9659 (0.9334–0.9995)
OR2 (>K)	0.9987 (0.9972–1.0002)	0.9990 (0.9981–0.9999)
Logarithmic likelihood ratio test *P*-value	.015	.054

Age, sex, race, education level, PIR, smoking behavior, drinking behavior, height, HbA1c, ALT, AST, COPD, triglyceride, total cholesterol, HDL-cholesterol, and LDL-cholesterol were adjusted.

ALT = alanine aminotransferase, AST = aspartate aminotransferase, CALLY index = C-reactive protein-albumin-lymphocyte index, CI = confidence interval, CKM = cardiovascular-kidney-metabolic, COPD = chronic obstructive pulmonary disease, HbA1c = hemoglobin A1c, HDL-cholesterol = high-density lipoprotein cholesterol, LDL-cholesterol = low-density lipoprotein cholesterol, OR = odds ratio, PIR = poverty-income ratio.

## 4. Discussion

Using data from a large, nationally representative cohort in NHANES, an inverse relationship was observed between the CALLY index and the likelihood of advanced CKM syndrome in US adults aged 20 years and older. Our analyses revealed that higher log-transformed CALLY index values corresponded to significantly lower odds of advanced CKM syndrome after comprehensive adjustment for demographic, socioeconomic, behavioral, and clinical confounders. Notably, when examined by tertiles, individuals in the highest CALLY group exhibited a 21% reduction in the risk of advanced CKM relative to those in the lowest tertile, underscoring the potential clinical relevance of this composite biomarker.

The inverse relationship identified between the CALLY index and advanced CKM syndrome highlights the important interplay among systemic inflammation, nutritional condition, and immune function in the development of cardiovascular, renal, and metabolic diseases.^[23]^ This index combines 3 key biological components: increased CRP serves as a marker of systemic inflammation, which is widely recognized as a contributor to endothelial damage, atherosclerosis progression, and worsening kidney injury^[24,25]^; serum albumin concentrations serve as a surrogate for nutritional status and overall physiological reserve, with hypoalbuminemia indicative of malnutrition and chronic catabolic states that exacerbate disease severity^[26,27]^; and lymphocyte count represents immune competence, wherein lymphopenia correlates with impaired immune surveillance and heightened vulnerability to chronic inflammation and infections.^[28,29]^ Collectively, these biomarkers integrate multidimensional systemic stressors implicated in the initiation and progression of CKM syndrome, thus providing a more holistic reflection of pathophysiological burden than any individual marker alone.

Previous biomarker investigations have traditionally focused on single or paired indicators related to inflammation (e.g., CRP and interleukins), nutrition (albumin and prealbumin), or immune parameters (lymphocyte subsets) to predict outcomes in CVD, CKD, or MS individually. While these studies have elucidated important mechanistic pathways and prognostic implications, they often fail to capture the complex interplay and synergism among these systems that underlie the convergence of these conditions in CKM syndrome. The application of the CALLY index, a composite measure integrating inflammatory, nutritional, and immunological dimensions, represents a novel advancement by encompassing the systemic milieu contributing to multiorgan dysfunction within an integrated syndrome framework.^[30,31]^ To the best of our knowledge, this investigation represents one of the early efforts to examine a combined biomarker encompassing inflammation, nutritional status, and immune function in CKM syndrome, offering preliminary findings that require confirmation through future prospective studies.

Furthermore, the nonlinear and age-specific associations observed in our analyses – specifically the stronger protective effect in younger adults – may stem from fundamental biological processes modulated by aging.^[32,33]^ In younger adults, the sharp inverse association below the inflection point could reflect higher physiological plasticity and greater responsiveness to improvements in inflammation, nutrition, and immune function. Conversely, as individuals age, chronic low-grade inflammation (“inflammaging”) and immunosenescence – characterized by altered immune cell function and reduced lymphocyte diversity – may diminish the sensitivity of systemic biomarkers, such as the CALLY index, to predict disease progression linearly. Additionally, older adults often experience nutritional vulnerability due to factors such as sarcopenia, comorbidities, and polypharmacy, complicating the direct interpretation of albumin levels.^[34–36]^ These age-related alterations likely contribute to the attenuation and linearization of the association in older populations, highlighting the nuanced and dynamic interplay between systemic physiology and CKM syndrome risk across the lifespan.^[37]^ This underscores the importance of incorporating age-specific considerations when deploying composite biomarkers such as the CALLY index in clinical and research settings.

From a clinical and public health perspective, the present findings position the CALLY index as a promising, accessible biomarker that integrates inflammatory status, nutritional health, and immune competence – 3 critical domains driving CKM syndrome pathophysiology.^[38]^ Given its reliance on routinely measured laboratory parameters (CRP, serum albumin, and lymphocyte count), the CALLY index offers a cost-effective and easily implementable tool for early risk stratification in diverse clinical settings. This simplicity enhances feasibility for widespread adoption in both primary care and specialty clinics, facilitating the identification of individuals at heightened risk for advanced CKM syndrome before overt clinical manifestation. Importantly, our data suggest that the utility of the CALLY index may be especially salient in younger populations, where nonlinear associations indicate substantial benefits derived from improvements in the biomarker components. Integrating the CALLY index into routine clinical and epidemiological assessments could enable healthcare providers to prioritize preventive interventions aimed at modulating systemic inflammation, optimizing nutritional status, and preserving immune resilience in at-risk individuals. Such stratified screening efforts would align with precision medicine paradigms, allowing earlier, tailored lifestyle, pharmacologic, or nutritional interventions aimed at halting or slowing CKM progression and reducing long-term morbidity.

Beyond risk stratification, the multidimensional nature of the CALLY index underscores the need for holistic disease management strategies that concurrently address inflammation, nutrition, and immune health. Interventions targeting only singular pathways may fail to mitigate the complex systemic burden characteristic of CKM syndrome. For instance, anti-inflammatory therapies could be complemented by nutritional optimization and strategies to enhance immune function, thereby tackling intersecting drivers of disease progression synergistically. The integration of the CALLY index into clinical workflows could support dynamic monitoring of therapeutic responses across these domains, guiding personalized adjustments in management plans. At the public health level, leveraging the simplicity and prognostic value of the CALLY index could inform population-based screening initiatives and health policy strategies aimed at reducing the growing burden of cardiometabolic and renal diseases. Early identification of vulnerable individuals through this composite biomarker could enable targeted resource allocation and preventive programs, ultimately curbing the incidence and progression of CKM syndrome and its attendant healthcare costs.

## 5. Strengths and limitations

A key strength of this study lies in its utilization of a large, nationally representative NHANES cohort, which supports the broader applicability of findings across the adult population in the United States. The thorough adjustment for numerous demographic, behavioral, and clinical confounders adds robustness to the reported association between the CALLY index and advanced CKM syndrome. Moreover, uncovering a nonlinear – and in specific subgroups, nearly linear – pattern offers fresh perspectives on the combined roles of systemic inflammation, nutrition, and immune status in influencing multisystem disease risk. Nonetheless, there are important limitations to acknowledge. The cross-sectional nature of the data prevents establishing temporal or causal links, restricting conclusions regarding the directionality between changes in the CALLY index and CKM syndrome development. Additionally, the absence of detailed information on critical lifestyle factors, including dietary habits, physical activity, and other behaviors, may introduce residual confounding. The possibility of unmeasured or imprecisely measured variables affecting the results cannot be excluded. Furthermore, reliance on biomarker data from a single time point may fail to capture relevant dynamic changes over the disease course. To better understand the causal mechanisms underlying the relationship between the CALLY index and CKM syndrome progression, future longitudinal research incorporating comprehensive behavioral, environmental, and clinical data is warranted. Such investigations would also be instrumental in validating the prognostic significance of the CALLY index and optimizing its potential role in clinical risk assessment and management strategies.

## 6. Conclusion

In this analysis of a large, nationally representative cohort of US adults, an inverse association emerged between the composite CALLY index and advanced CKM syndrome. These results emphasize the potential utility of combining markers of systemic inflammation, nutritional status, and immune function into a single composite measure that reflects the complex multisystem nature of CKM syndrome. Notably, the observed nonlinear relationships, particularly among younger individuals, highlight critical windows for early identification and intervention. Due to its relative simplicity, low cost, and broad applicability, the CALLY index holds considerable promise as a pragmatic tool for refining risk stratification, informing personalized prevention strategies, and guiding holistic management approaches that address intertwined cardiometabolic and renal dysfunction. Nonetheless, additional prospective research is necessary to confirm these findings and further clarify the biological mechanisms and clinical relevance of modulating the factors represented by the CALLY index. Ultimately, integrating such composite biomarkers into clinical and public health frameworks may substantially advance precision medicine efforts aimed at mitigating the escalating burden of CKM syndrome globally.

## Author contributions

**Conceptualization:** Yi Liu.

**Methodology:** Yi Liu.

**Data curation:** Yi Liu.

**Formal analysis:** Yi Liu.

**Investigation:** Yi Liu.

**Software:** Yi Liu.

**Visualization:** Yi Liu.

**Supervision:** Yong Li.

**Writing – original draft:** Yi Liu.

**Writing – review & editing:** Yong Li.

## Supplementary Material
