# Supplementary material for: Age-related associations between the CALLY index and advanced cardiovascular-kidney-metabolic (CKM) syndrome: Insights from NHANES population data
Source: Medicine (Baltimore). 2026 Mar 13;105(11):e47718. doi: 10.1097/MD.0000000000047718 (PMC12991715; doi:10.1097/MD.0000000000047718)
Supplement: Supplementary file 1 [file medi-105-e47718-s001.docx]

**Supplementary Table 1. Definitions of CKM Syndrome Stages.**

| **CKM**  **syndrome stages** | **Definition** |
| --- | --- |
| Stage 0 | Normal BMI (<25 kg/m^2^ or <23 kg/m^2^ if Asian ethnicity);  Normal waist circumference (<88/102 cm in women/men or <80/90 cm in women/men if Asian ethnicity);  Normoglycemia;  Normotension;  Normal lipid status;  No evidence of CKD;  No evidence of CVD. |
| Stage 1 | Elevated BMI (≥25 kg/m^2^ or ≥23 kg/m^2^ if Asian ethnicity);  Elevated waist circumference (≥88/102 cm in women/men or ≥80/90 cm in women/men if Asian ethnicity);  Prediabetes (fasting blood glucose between 100 mg/dL and 126 mg/dL or glycated hemoglobin between 5.7% and 6.4%). |
| Stage 2 | Elevated fasting serum triglycerides (≥135 mg/dL);  Hypertension (blood pressure ≥140/90 mmHg, self-reported history of hypertension, or current antihypertensive treatment);  Diabetes (fasting blood glucose ≥126 mg/dL, glycated hemoglobin≥ 6.5%, self-reported history of diabetes, or current hypoglycemic treatment);  Metabolic syndrome (≥3 of the following: elevated waist circumference,  high density lipoprotein cholesterol <40 mg/dL for men and <50 mg/dL for women, fasting serum triglycerides ≥ 150 mg/dL, blood pressure ≥ 130/80 mmHg and/or current antihypertensive treatment, fasting blood glucose ≥100 mg/dL);  Moderate-to-high-risk CKD^1^. |
| Stage 3 | Very-high-risk CKD^1^;  High predicted 10-year CVD risk^2^. |
| Stage 4 | Self-reported established CVD (coronary heart disease, angina, heart attack, heart failure, and stroke). |

^1^CKD stages were identified based on GFR and urinary albumin-to creatinine ratio.

^2^The 10-year CVD risk was calculated using the Framingham risk calculation based on the Framingham risk score.

Abbreviations: BMI, Body Mass Index; CKD, chronic kidney disease; CKM, Cardiovascular- Kidney-Metabolic; CVD, cardiovascular disease.

**Supplementary Table 2. Full multivariable logistic regression analysis of the association between CALLY index tertiles and advanced CKM syndrome (Model 3).**

| Variable | OR (95% CI) | P-value |
| --- | --- | --- |
| **CALLY index tertiles** |  |  |
| Tertile 1 (Low) | Reference |  |
| Tertile 2 (Middle) | 0.83 (0.72, 0.96) | 0.0117 |
| Tertile 3 (High) | 0.79 (0.67, 0.93) | 0.0048 |
| **Age** (per year) | 1.13 (1.12, 1.14) | <0.0001 |
| **Sex** | 0.59 (0.49, 0.72) | <0.0001 |
| **Race/Ethnicity** |  |  |
| Non-Hispanic White | Reference |  |
| Mexican American vs Ref | 0.58 (0.48, 0.71) | <0.0001 |
| Non-Hispanic Black vs Ref | 1.30 (1.09, 1.54) | 0.0029 |
| Other races vs Ref | 0.69 (0.55, 0.87) | 0.0015 |
| **Education level** |  |  |
| Some college or above | Reference |  |
| High school vs Ref | 1.07 (0.91, 1.26) | 0.3879 |
| Less than high school vs Ref | 0.85 (0.72, 1.00) | 0.0443 |
| **PIR** | 0.85 (0.82, 0.90) | <0.0001 |
| **Smoking status** |  |  |
| Never | Reference |  |
| Former vs Ref | 1.09 (0.94, 1.26) | 0.2476 |
| Current vs Ref | 1.88 (1.56, 2.27) | <0.0001 |
| **Alcohol Use** |  |  |
| Never | Reference |  |
| Former vs Ref | 1.24 (1.06, 1.45) | 0.0065 |
| Mild vs Ref | 1.08 (0.86, 1.35) | 0.5121 |
| Moderate vs Ref | 1.03 (0.85, 1.26) | 0.7457 |
| Heavy vs Ref | 0.98 (0.77, 1.24) | 0.8604 |
| **Height (cm)** | 0.99 (0.98, 1.00) | 0.023 |
| **HbA1c (%)** | 1.19 (1.13, 1.26) | <0.0001 |
| **ALT (IU/L)** | 1.00 (1.00, 1.00) | 0.04 |
| **AST (IU/L)** | 1.00 (0.99, 1.00) | 0.0699 |
| **COPD (Yes vs No)** | 1.47 (1.17, 1.84) | 0.001 |
| **Triglyceride (mg/dL)** | 1.00 (1.00, 1.00) | 0.8674 |
| **Total cholesterol (mg/dL)** | 1.00 (1.00, 1.01) | 0.6566 |
| **HDL-cholesterol (mg/dL)** | 0.98 (0.97, 0.98) | <0.0001 |
| **LDL-cholesterol (mg/dL)** | 0.99 (0.98, 1.00) | 0.0004 |
